# Comparison of the clinical efficacy of a femoral neck system versus cannulated screws in the treatment of femoral neck fracture in young adults

**DOI:** 10.1186/s12891-021-04888-0

**Published:** 2021-11-29

**Authors:** Changjun He, Yao Lu, Qian Wang, Cheng Ren, Ming Li, Mingyi Yang, Yibo Xu, Zhong Li, Kun Zhang, Teng Ma

**Affiliations:** 1 Department of Orthopaedic Surgery, HongHui Hospital, Xi’an Jiaotong University, 555Youyi East Road, Xi’an 710054, Shaan’xi Province China; 2 Yan’an University, Yan’an, 710000 Shaanxi China

**Keywords:** Femoral neck fracture, Femoral neck system, Cannulated screw, Internal fixation

## Abstract

**Background:**

To compare the clinical efficacy of a femoral neck system (FNS) and cannulated screws (CS) in the treatment of femoral neck fracture in young adults.

**Methods:**

Data from 69 young adults, who were admitted for femoral neck fracture between March 2018 and June 2020, were retrospectively analyzed. Patients were divided into two groups according to surgical method: FNS and CS. The number of intraoperative fluoroscopies, operative duration, length of hospital stay, fracture healing time, Harris score of hip function, excellent and good rate of hip function, and postoperative complications (infection, cut out the internal fixation, nail withdrawal, and femoral neck shortening) were compared between the two groups. Hip joint function was evaluated using the Harris Hip Scoring system.

**Results:**

All 69 patients had satisfactory reduction and were followed up for 12–24 months, with a mean follow-up of 16.91 ± 3.01 months. Mean time to fracture healing was13.82 ± 1.59 and 14.03 ± 1.78 weeks in the FNS and CS groups, respectively. There was a statistical difference in the number of intraoperative fluoroscopies between the 2 groups (*P* = 0.000). There were no significant differences, in operation duration, hospital length of stay, fracture healing time, complications, Harris Hip Score for hip function and excellent and good rate between the two groups (*P* > 0.05). The incidence of complications was 6.1%(2/33) in the FNS group lower than 25%(9/36) in the CS group, a difference that was statistically significant (*P* = 0.032). At the last follow-up, the Harris Hip Score of the hip joint in the FNS group was 90.42 ± 4.82and 88.44 ± 5.91 in the CS group.

**Conclusions:**

Both treatment methods resulted in higher rates of fracture healing and excellent hip function. Compared with CS, the FNS reduced the number of intraoperative fluoroscopies, radiation exposure to medical staff and patients, and short-term complications including femoral neck shortening and bone nonunion.

## Background

Femoral neck fractures occur mainly in elderly individuals, accounting for 48–54% of hip fractures, while younger adults (age < 65 years) who experience femoral neck fracture account for approximately 3% of cases; however, the treatment of younger adults is a challenge in traumatic orthopedics [1–3]. A consensus has been reached regarding the surgical treatment of femoral neck fracture in young adults. Commonly used internal fixation surgical methods involve the use of hollow compression screws, dynamic hip screws, compression plate(s), and new compression locking nail plate systems. However, due to problems with loosening of internal fixation devices, nail back, and weak anti-rotation force(s) in all types of methods, it remains controversial which internal fixation method is currently the best [4]. In an autopsy study involving cadaver specimens, Putnam et al. [5] reported that the inferior retinacular artery of the medial femoral artery is an important source of blood to the femoral head after femoral neck fracture, which is easily injured in the process of high-energy impact(s) and corrective surgery itself, and can lead to serious postoperative complications including bone nonunion and necrosis of the femoral head, among others, with an incidence of approximately 7–33% and 10–30% [6]. The severity of postoperative complications has prompted many physicians to reconsider how to reduce injury through minimally invasive implantation and reduce complications during surgery. In recent years, DePuy Synthes (Johnson & Johnson Medical Devices, New Brunswick, NJ, USA) developed a new internal fixation device for the treatment of femoral neck fractures in young adults known as the “Femoral Neck System” (FNS). However, its clinical efficacy compared with traditional internal fixation is unknown. Therefore, this study aimed to compare the clinical efficacy of the FNS versus cannulated compression screws (CS) in the treatment of femoral neck fracture in young adults.

## Methods

### Patients

This study was approved by the Ethics Committee of the HongHui Hospital Affiliated to Xi’an Jiao tong University (Xi’an, Shaanxi, China; 202,104,006). A total of 69 young adult patients, who experienced femoral neck fracture and admitted between March 2018 and June 2020, were included. All patients provided informed consent prior to participation in the study.

### Inclusion criteria and exclusion criteria

All patients underwent surgical treatment and follow-up. Inclusion criteria were as follows: age, 18–65 years; imaging diagnosis of femoral neck fracture; FNS or 3 CSs were used for fixation; and availability of complete case data. Individuals who underwent non-surgical treatment, those with old or pathological fractures, necrosis of the femoral head, long-term heavy alcohol use, or the use of hormone drugs were excluded.

The 69 patients were divided into two groups according to the surgical method: FNS and CS. Patients were treated with FNS or CS internal fixation. Sex, age, fracture type, cause of injury, were compared between the two groups, (Table 1).

**Table 1:** Comparison of general characteristics between the two groups

Characteristic	Group		χ^**2**^/t value	***P*** value
	Femoral neck system	Cannulated screws		
Sex				
Male	18 (54.5)	22 (61.1)	0.305	0.581
Female	15 (45.5)	14 (38.9)		
Age (years)	50.61 ± 10.30	47.58 ± 10.31	1.217	0.228
Garden classification				
Type I	1 (3.0)	2 (5.6)	0.006	0.939
Type II	8 (24.2)	9 (25.0)	0.005	0.942
Type III	19 (57.6)	20 (55.6)	0.029	0.866
Type IV	5 (15.2)	5 (13.9)	0.037	0.847
Cause of injury				
Motor vehicle collision	12 (36.4)	11 (30.6)	0.261	0.609
Fall(s)	21 (63.6)	25 (69.4)		

Data are presented as n (%) or mean standard ± deviation unless otherwise indicated

### Surgical method

After general anesthesia or a satisfactory level of anesthesia administered via the spinal canal, all patients were positioned with a affected hip elevation of 10°–15° while supine on a traction table, with appropriate traction for closed reduction of the fracture. According to Garden index, namely, anteroposterior and lateral radiograph X-ray. The angle between the axial line of the femoral head trabecula and the medial cortex of the femoral shaft was 160–180° on the anteroposterior radiograph and 180° on the lateral film as far as possible, with forward tilt < 5° and backward tilt < 10° [7, 8]. If closed reduction was not ideal, open reduction and internal fixation was performed. A Smith-Peterson incision was used and T-shape incision was used to open the joint capsule, and the fractured end of the femoral neck was exposed.

**Fig. 1:**
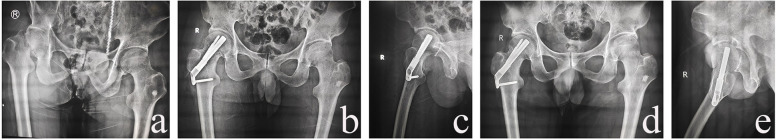
A 53-year-old male with right femoral neck fracture (Garden type III) in the Femoral Neck System (FNS) group. **a** Anteroposterior X-ray images before surgery; **b**, **c** postoperative anteroposterior and lateral X-ray revealing satisfactory reduction of the fracture and good FNS position; **d**, **e** anteroposterior and lateral radiographs 3 months postoperatively revealing fracture healing and good internal fixation

For patients in the FNS group, an anti-rotation Kirschner wire was first placed to maintain fracture reduction. The anti-rotation Kirschner wire was positioned as close to the upper margin of the femoral neck as possible, and the lateral position was as far as away as possible from the center of the femoral neck to avoid affecting placement of the FNS power rod. Then, a guide needle was inserted in the direction of the femoral neck with the assistance of a guide device. The guide needle was positioned as near to the center of the femoral neck as possible. After a satisfactory position was achieved, the bone marrow canal was opened along the guide needle and the FNS power rod was gently inserted. After satisfactory attachment of the lateral plate of the femur, 1–2 locking screws were inserted after drilling along the guide system. Finally, the anti-rotation screw channel of the guide was opened, and an anti-rotation screw of the appropriate length was inserted. The position of the FNS was confirmed fluoroscopically, and the anti-rotation Kirschner wire was removed after satisfactory positioning was confirmed. After checking the instruments to confirm the absence of errors, the wound was rinsed and sutured layer by layer and bandaged using sterile dressing. A representative X-ray of one case after FNS internal fixation is presented in Fig. 1.

**Fig. 2:**
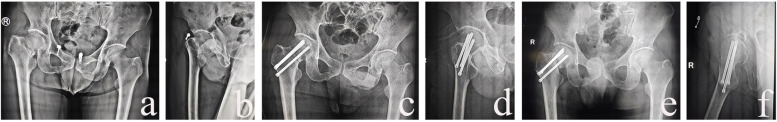
A 50-year-old male with right femoral neck fracture (Garden type III) in the cannulated screw (CS) group. **a**, **b** Anteroposterior and lateral X-ray images before surgery; **c**, **d** postoperative anteroposterior and lateral radiographs revealing satisfactory reduction of the fracture. Lateral radiographs indicating poor placement of hollow nails; **e**, **f** anteroposterior and lateral radiographs 12 months after surgery. The fracture was not healed

**Fig. 3:**
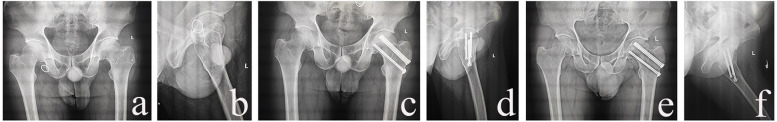
A 41-year-old male with left femoral neck fracture (Garden type II) in the cannulated screw (CS) group. **a**, **b**. Anteroposterior and lateral X-ray images before surgery; **c**, **d** postoperative anteroposterior and lateral radiographs revealing satisfactory reduction of the fracture. Location satisfactory of hollow nails; **e**, **f** anteroposterior and lateral radiographs 3 months after surgery revealing fracture healing is good

For patients in the CS group, the first guide needles was placed under fluoroscopy and the direction of the guide needles was adjusted and the tip was position 5 mm below the cartilage. The guide wire in the lateral X-ray was located in the center of the femoral neck and tilted forward by approximately 15°. Then, the back above and front above of the first guide needle parallel placement of the second, the third guide needle. The position of the 3 guide pins was in an inverted triangle; otherwise, guide pin position was adjusted accordingly. After the position of the guide wire was deemed satisfactory, a small incision was made at the end of the guide wire, and three CS were inserted along the direction of the guide wire to fix the fracture. The internal fixation position was examined in anteroposterior and lateral perspective views to confirm that the internal fixation position and fracture fixation were satisfactory. After checking the instruments, washed and sutured the wound and bound with sterile dressing. A representative X-ray of one case of nonunion after CS internal fixation is presented in Fig. 2. A representative X-ray of one case after CS internal fixation is presented in Fig. 3.

### Postoperative management

Antibiotics were administered within 24 h after surgery to prevent infection, and heparin sodium was administered subcutaneously to prevent deep vein thrombosis. The next day, radiography and computed tomography were performed to determine reduction and internal fixation. Patients were instructed to exercise the muscles of the affected limb, avoid straight leg raises and weight-bearing activities, and mainly rest in bed. At 1, 2, 3, 6, and 12 months after surgery, anteroposterior and lateral X-rays of both hips were reexamined to assess fracture healing, and initiation of partial or total weight bearing was determined accordingly.

### Observation index and evaluation of curative effect

The number of intraoperative fluoroscopies, operative duration, length of hospital stay, fracture healing time, Harris Hip Score for hip function, excellent and good rate of hip function (That is, the proportion of excellent and good in Harris hip Score.), and postoperative complications (infection, cut out the internal fixation, nail withdrawal, and femoral neck shortening) were compared between the two groups. Hip function was evaluated using the Harris Hip Scoring system [9], which includes four domains: pain, function, degree of deformity, and range of motion of the joint. At the same time, in accordance with the Harris Hip Scoring system, clinical efficacy was graded as follows: 90–100, excellent; 80–89, good; 70–79, moderate; and < 70, poor.

### Statistical analysis

Statistical analysis was performed using SPSS version 23.0 (IBM Corporation, Armonk, NY, USA). The Shapiro-Wilk test was first used to determine whether the data were normally distributed. Among the variables, age, operative duration, number of intraoperative fluoroscopies, hospital stay, fracture healing time, and Harris Hip Score were normally distributed, and homogeneity of variance is expressed as mean ± standard deviation (SD). The independent-samples *t*-test was used to compare the two groups. Enumerative data, such as sex, excellent and good rates, and complications, are expressed as rate (%) and were tested using the chi-square (χ^2^) test. When the theoretical frequency was < 1, Fisher’s exact probability method was used to test. Differences with *P* < 0.05 were considered to be statistically significant.

## Results

Preoperative baseline characteristics of the patients, including sex, age, fracture type, and cause of injury are summarized in Table 1. The mean age of patients comprising the FNS and CS groups was 50.61 ± 10.30and 47.58 ± 10.31 years, respectively. Male patients in the FNS group accounted for 54.5%(18/33), female patients accounted for 45.5%(15/33); in the CS group, male patients accounted for 61.1%(22/36) and female patients accounted for 38.9%(14/36). Garden III was the main fracture type in the FNS and CS groups, accounting for 57.6%(19/33) and 55.6%(20/36), respectively. Falls were the main cause of injury in both groups, accounting for 63.6%(21/33) and 69.4%(25/36) in the FNS and CS groups, respectively. There were no significant differences in sex, age, fracture type, and cause of injury between the two groups (*P* > 0.05).

Patients in both groups had satisfactory reduction after surgery. The patients were followed up for 12–24 months, with a mean follow-up of 16.91 ± 3.01 months. The average operation duration in the FNS group was 49.94 ± 14.46 min, which was less than that in the CS group (56.11 ± 12.48 min); the difference, however, was not statistically significant(*P* = 0.062). The average number of intraoperative fluoroscopies in the FNS group was 10.58 ± 1.89, which was less than that in the CS group (18.33 ± 3.82); this difference was statistically significant (*P* = 0.000) (Table 2). There was no statistically significant difference in hospital length of stay, postoperative fracture healing time between the two groups (*P*>0.05) (Table 2). Femoral neck shortening occurred in 1 patient in the FNS group during follow-up and in 3 patients in the CS group. Cut out the internal fixation occurred in 1 patient in the FNS group and in 2 patients in the CS group. Nails were withdrawn in 1 patient 2 months after surgery, bone nonunion occurred in 2 patients, and no obvious callus formation was found at the fracture end, nail retreat in 1 patient in the CS group. Clinical bone healing was achieved in the remaining patients in both groups. The incidence of complications was 6.1% (2/33) in the FNS group lower than 25% (9/36) in the CS group; the difference was statistically significant (*P* = 0.032). At the last follow-up, there was no statistically significant differences in hip closure function score and excellent and good rate between the two groups (*P* > 0.05) (Table 2). The excellent and good rate of the hip joint function in the FNS group was 90.9% (30/33) and 88.9% (32/36) in the CS group. The Harris Hip Score of the hip joint in the FNS group was 90.42 ± 4.82and 88.44 ± 5.91 in the CS group.

**Table 2:** Comparison of perioperative period and postoperative conditions between the two groups

Variable	Group			
	Femoral neck system (***n*** = 33)	Cannulated screws (***n*** = 36)	χ2/t value	***P*** value
Operation duration (min)	49.94 ± 14.46	56.11 ± 12.48	−1.902	0.062
Fluoroscopies, n	10.58 ± 1.89	18.33 ± 3.82	−10.547	0.000
Hospital length of stay (days)	5.12 ± 1.88	4.78 ± 1.55	0.830	0.410
Healing time (weeks)	13.82 ± 1.59	14.03 ± 1.78	−0.514	0.609
Harris Hip Score	90.42 ± 4.82	88.44 ± 5.91	1.517	0.134
Excellent and good rate	30 (90.9)	32 (88.9)	0.015	0.903
Complications	2 (6.1)	9 (25.0)	4.609	0.032
Nonunion of bone	0 (0)	2 (5.6)		
Nail retreat	0 (0)	2 (5.6)		
Femoral neck shortening	1 (3.0)	3 (8.3)		
Cut out the internal fixation	1 (3.0)	2 (5.6)		

Data are presented as n (%) or mean ± standard deviation unless otherwise indicated

## Discussion

With the rapid advances in societies and economies, traumatic injuries due to various causes are constantly increasing, and the rate of femoral neck fracture is also gradually increasing [10]. Although hip arthroplasty has been widely used in the treatment of femoral neck fracture in elderly individuals, it is not necessarily the best treatment for younger patients [11]. Closed reduction and internal fixation can mitigate the disruption of blood supply to the femoral head, and is the preferred treatment for younger adults with femoral neck fracture; nevertheless, some patients require open reduction and internal fixation [12]. There are various methods of internal fixation; however, none can completely prevent the occurrence of complications such as nonunion of fracture, femoral neck shortening, and necrosis of the femoral head.

In recent years, the CS method has been widely used in the treatment of femoral neck fractures in young adults owing to its minimally invasive fixation, low cost of materials, good anti-rotation force, and ability to adequately address most femoral neck fractures. However, for unstable femoral neck fracture at Pauwels angle > 50°, its biomechanical properties are poor [13, 14]. After the emergence of the FNS in recent years, Stofel et al. [15] compared and studied the biomechanical properties of Pauwels III type femoral neck fracture treated using FNS, dynamic hip screw, and 3 CS. The results revealed that differences in the biomechanical properties of the FNS and dynamic hip screw fixation were not statistically significant, but they were all stronger than that of 3 CS fixation, with a difference that was statistically significant (*P* < 0.05). A biomechanical study of the FNS by Schopper et al. [16] reached the same conclusion as Stofel et al., indicating that FNS is a reliable internal fixation method with similar biomechanical characteristics. The FNS features simple operation, low-level trauma, angular stability, and a good anti-rotation effect. One of the authors of the present study has previous experience with dynamic hip screw surgery and the short learning curve. FNS uses a nail-in-nail combination to increase anti-rotation stability and overall biomechanical properties, and the FNS power bar is a percussion placement rather than rotational placement of a dynamic hip head nail, avoiding the secondary rotational displacement of the femoral head caused by rotational torque [17]. The CS method requires three screws to be parallel with one another and to be in an inverted triangle formation, and requires a higher spatial distribution of screws. Therefore, placement of the guide wire needs to be adjusted repeatedly; however, too many adjustments will exacerbate destruction of blood supply to the femoral head and increase the number of required intraoperative fluoroscopies, which increases X-ray radiation exposure to medical staff and patients. In this study, the mean number of intraoperative fluoroscopies in the FNS group was 10.58 ± 1.89, which was lower than that in the CS group (18.33 ± 3.82), and the difference was statistically significant (*P* = 000). In addition, the mean operation duration in the FNS group was 49.94 ± 14.46 min, which was shorter than that of the CS group (56.11 ± 12.48 min); however, the difference was not statistically significant (*P* = 0.062). Although the effect of operation duration on the femoral head remains controversial, shorter operation duration is definitely beneficial to patients.

According to a previous study, the nonunion rate of femoral neck fracture treated with 3 CS alone was as high as 19%, and the nonunion rate of fracture treated with angle-stabilized internal fixators was 8% [12, 18]. In this study, 1 patient in the CS group presented with nail retreat and discomfort in the affected hip 2 months after surgery, while 2 others experienced non-union of the fracture, 1 case had screw withdrawal. The remaining patients in the two groups achieved bone union, and the rate of non-union of the fracture was 8.3% (3/36), which was lower than previous reports. This may be explained by the smaller number of patients, a fracture end sliding compression effect, and stability of the fixation. Postoperative nonunion of femoral neck fractures seriously affects patient prognosis [19]. There are many factors that lead to such complications, but only a few of which are amenable to artificial intervention, such as the quality of fracture reduction and the timing of postoperative weight-bearing activities. Studies have shown that the quality of fracture reduction is the main factor affecting the postoperative efficacy of fracture repair [20]. Therefore, surgical reduction should be performed in strict accordance with the Garden reduction index. For patients with unsatisfactory reduction, open reduction should be performed in a timely manner, and internal fixation should be performed after anatomical reduction as far as possible. Anatomical reduction is not only beneficial in protecting blood supply to the femoral head, but also plays a crucial role in the recovery of biomechanical factors of the femoral neck. Premature postoperative weight-bearing activity is another factor that affects fracture healing. In vitro studies have shown that only 25% of the stress of a femoral neck fracture can be sustained by internal fixation after surgery, and 75% of the stress is borne by the fracture itself [21]. Therefore, even if a crutch is not weight-bearing, because of the muscle to maintain body balance, the fracture will bear greater stress, thus increasing the risk for fracture nonunion. Osteoporosis caused by prolonged bed rest is also not conducive to fracture healing; it has been reported that the rate of fracture nonunion in patients with osteoporosis can reach 28% [22]. Therefore, although it is necessary to engage in postoperative rehabilitation, it is important to be cautiously aware of the timing and extent of these activities.

In the present study, patients in both the FNS and CS groups exhibited shortening of the femoral neck, although no obvious clinical symptoms were exhibited by either. There are two types of healing methods for femoral neck fracture after internal fixation: shortening union and non-shortening union. Related research has reported that the incidence of postoperative femoral neck shortening with hollow screws is approximately 17–65%, and femoral neck shortening and hip joint function has a strong correlation [23, 24]; at the same time, the femoral neck shortening and internal fixation are also easy to cut out the femoral head, aggravating the functional damage of the hip joint. In this study, 1 patient in the FNS group and 2 patients in the CS group had internal fixation excision, but there was no obvious discomfort at present. As such, complications should concern surgeons. To ensure fracture healing, most implants are designed to allow for a range of sliding compression at the fracture end. Among these, the FNS has a 20 mm sliding compression space to facilitate contact of the fracture end and prevent bone absorption at the fracture end, resulting in bone nonunion. This space is part of the power rod sleeve, which controls the direction of sliding pressure, thus increasing internal resistance and mitigating shortening of the femoral neck. In contrast, hollow screws do not have this advantage. The strength of fracture fixation is proportional to the strength of the bone, and patients with osteoporosis or poor cortical bone are more likely to experience excessive compression and bone absorption at the fracture end in the early stage(s) of fracture healing, leading to femoral neck shortening after fracture healing [23]. Other studies have reported that postoperative femoral neck shortening is mainly related to characteristics of the anatomical structure and the mechanical environment [25, 26]. CS lack reliable and effective fixation of the femoral side and, once shortening of the femoral neck occurs, the nail will retreat. If serious, nail retreat will result in patient discomfort and increase the number of medical visits. However, this does not occur with the FNS because there is plate screw fixation on the femoral side, and the cross-fixation angle between the anti-rotation screw and the power bar provides angular stability and prevents the occurrence of nail retreat [15, 16].

The present study had limitations, the first of which were its retrospective, single-center design and small sample size; as such, our conclusions are contingent. Therefore, larger-scale, prospective, randomized, case-control studies are needed to comprehensively evaluate the effectiveness and safety of the FNS method. Second, the incidence of femoral head necrosis, secondary revision surgery, and long-term functional outcomes were not compared during the short follow-up period.

## Conclusion

We compared the FNS and CSs in the treatment of femoral neck fractures in young adults. The results revealed no significant differences in fracture healing time and hip function between the two methods, and both methods resulted in good postoperative hip function. The use of the FNS for internal fixation can reduce the number of intraoperative fluoroscopies, X-ray radiation exposure of medical personnel and patients, and the incidence of complications such as bone nonunion, femoral neck shortening, and nail retreat. However, this was only a preliminary study, and results need to be demonstrated over a longer follow-up and larger sample size.

## Data Availability

All data analyzed in this study has been provided in the manuscript.

## Abbreviations

FNS: Femoral Neck System
CS: Cannulated screw(s)
